# Supplementary material for: The relationship between object-based spatial ability and virtual navigation performance
Source: PLoS One. 2024 May 9;19(5):e0298116. doi: 10.1371/journal.pone.0298116 (PMC11081363; doi:10.1371/journal.pone.0298116)
Supplement: S1 Checklist — (DOCX) [file pone.0298116.s001.docx]

STROBE Statement—checklist of items that should be included in reports of observational studies

|  | Item No. | Recommendation | Page No. | Relevant text from manuscript |
| --- | --- | --- | --- | --- |
| **Title and abstract** | 1 | (*a*) Indicate the study’s design with a commonly used term in the title or the abstract | 4 | “Here, we report the use of an established mobile gaming app, Sea Hero Quest (SHQ), as a measure of navigation ability in a sample of young, predominantly female university students (N = 78; 20; female = 74.3%; mean age = 20.33 years).” |
|  |  | (*b*) Provide in the abstract an informative and balanced summary of what was done and what was found | 4 | “Here, we report the use of an established mobile gaming app, Sea Hero Quest (SHQ), as a measure of navigation ability in a sample of young, predominantly female university students (N = 78; 20; female = 74.3%; mean age = 20.33 years). We used three separate tests of navigation embedded in SHQ: wayfinding, path integration and spatial memory in a radial arm maze. In the same participants, we also collected measures of mental rotation (Mental Rotation Test), visuospatial processing (Design Organization Test) and visuospatial working memory (Digital Corsi). We found few strong correlations across our measures. Being good at wayfinding in a virtual navigation test does not mean an individual will also be good at path integration, have a superior memory in a radial arm maze, or rate themself as having a strong sense of direction. However, we observed that participants who were good in the wayfinding task of SHQ tended to perform well on the three visuospatial tasks examined here, and to also use a landmark strategy in the radial maze task.” |
| Introduction | | | |  |
| Background/rationale | 2 | Explain the scientific background and rationale for the investigation being reported | 5-6 | Rationale and existing literature are stated in the introduction section.  The following paragraph from the aforementioned section explains why we are interested in navigation and related abilities in this study: “Navigation is a fundamental skill that underlies exploration and survival. The ability to effectively navigate involves a host of capacities such as planning routes, reading maps, recognizing landmarks and keeping track of direction. Deficits in these competencies may constitute an early marker of degenerative conditions such as Alzheimer’s disease (AD); furthermore, spatial abilities are impaired in various neurological disorders or conditions, such as multiple sclerosis, vestibular syndromes and autism ([1-4](#_ENREF_1)). Furthermore, navigation impairments like disorientation can affect one’s quality of life by causing distress and impediments in daily functioning ([5](#_ENREF_5), [6](#_ENREF_6)). Therefore, understanding individual differences in competencies and strategies underlying navigation can be useful in not only identifying risk factors of cognitive decline, but also in designing tools, interventions and environments to cater to unique navigational needs. More broadly, knowledge of the complex mechanisms and interactions involved in navigation will advance the domain of spatial cognition at large.”  The paragraphs below from the introduction section explains why we specifically want to use Sea Hero Quest as a measure of navigation ability:  “Creating a valid standardised test of navigation is not easy. This is because of the difficulties in achieving the required levels of environmental manipulation and experimental control in standard research settings, which are further compounded by the problem of testing large enough cohorts to account for the wide variations in performance. In recent years, virtual reality (VR) and the widespread touch-screen technology on tablet and mobile devices have offered new possibilities for testing. Our team capitalised on these possibilities by developing a set of tests for navigation ability in the form of the video game app Sea Hero Quest (SHQ) ([7](#_ENREF_7)). We have employed SHQ to test the navigation ability of 3.9 million people across the world ([8](#_ENREF_8), [9](#_ENREF_9)). SHQ has good test-retest reliability ([10](#_ENREF_10)), and has been shown to be predictive of real-world navigational performance ([11](#_ENREF_11)).  Studies using SHQ have revealed that gender differences in navigation ability for a country can be partially attributed to gender inequality ([8](#_ENREF_8)). They have also found that individuals are more adept at navigating environments that are topologically similar to those in which they were raised ([12](#_ENREF_12)). Such studies have also shown that performance is best for older participants who report sleeping 7 hours per night compared to those reporting more or less sleep ([13](#_ENREF_13)). It has also been used in the study of AD, for instance, to detect sub-optimal navigation performance in pre-clinical AD, to classify spatial impairments in healthy participants at a high risk of AD ([14](#_ENREF_14)), and to detect those AD patients most prone to disorientation ([15](#_ENREF_15)). Finally, SHQ has also been employed to detect wayfinding and path integration (PI) deficits in patients with traumatic brain injury ([16](#_ENREF_16)).” |
| Objectives | 3 | State specific objectives, including any prespecified hypotheses | 8 | The last paragraph of the introduction lists the objectives and hypotheses we explore in this study, which is as follows:  “In this study, we explore how visuospatial abilities, navigation strategy and gameplay stress relate to performance on SHQ and to each other. Based on the literature (e.g., Wolbers & Hegarty ([17](#_ENREF_17))), we made 20 predictions. We expected wayfinding to correlate with other measures due to its diverse demands such as perception, memory, decision-making, etc. Specifically, we predicted that longer duration to complete wayfinding levels (i.e., wayfinding inefficiency) would be associated with lower mental rotation, visuospatial processing, VSWM, sense of direction and mapping tendency as measured by the MRT, DOT, D-Corsi, SBSOD and NSQ, respectively. Greater wayfinding inefficiency would also be related to higher total SHQ gameplay duration and SHQ stress. We further predicted that more correct answers on PI levels would be associated with stronger VSWM and sense of direction as measured by D-Corsi and SBSOD, respectively. Moreover, we hypothesized that the number of reference memory errors (i.e., RM errors) and spatial working memory errors (i.e., SWM errors) on RAM levels would positively correlate with each other and negatively with D-Corsi and SBSOD scores. Additionally, we predicted that mental rotation and visuospatial processing, as measured by the MRT and the DOT, respectively, would positively correlate with each other and with VSWM, as measured by the D-Corsi. Our final hypothesis was that sense of direction and mapping tendency, as measured by the SBSOD and the NSQ, respectively, would positively correlate with each other. Table 1 below shows the 20 hypothesised relationships between performance on different tasks.”  Table 1 in the introduction section lists all the 20 hypothesised relationships between performance on different tasks. |
| **Methods** |  |  |  |  |

| Study design | 4 | Present key elements of study design early in the paper | 12-16 | The key elements of study design are presented in the Methods section.  “2.3 Sea Hero Quest Tasks  2.3.1 Sea Hero Quest App ([7](#_ENREF_7), [8](#_ENREF_8), [45](#_ENREF_45))  SHQ is a VR navigation game for mobile and tablet devices in which participants are required to navigate a three-dimensional environment of water bodies. Navigation abilities of participants are assessed through three types of levels: Wayfinding, PI and RAM (see Fig 1). To avoid a ceiling effect, participants played the somewhat “difficult” levels of SHQ. The difficulty of wayfinding levels was based on the number of goals in a particular level, and how far apart they were located from each other. Levels in which there were four or more goals that were located at a considerable distance from each other were considered difficult. For PI levels, difficulty was determined by the number of turns participants had to take to get from the starting point to the final destination. Levels in which there were at least four turns were selected. Based on the aforementioned criteria, we chose levels 16, 37, 32 and 43 for wayfinding to keep a broader selection of levels in terms of spatial characteristics of the environment. For PI, we selected levels 44, 49, 54, 59, 64, 69 and 74 that were later in the game and more challenging. For the purpose of this study, we only analysed levels that were played by all participants in the original trauma study. Accordingly, we considered levels 16 and 37 for wayfinding, and levels 44, 49, 54 and 74 for PI. Lastly, participants played all the RAM levels as there are only five such levels in SHQ.      In wayfinding levels, participants are required to navigate to checkpoints in an ordered manner based on a map presented to them before the game begins. Performance on wayfinding levels was operationalised as the average inefficiency across all the levels. Lower inefficiency values indicate better wayfinding performance, meaning that participants covered less distance to complete all the levels. To control for prior video gaming experience, we standardised wayfinding inefficiency by dividing it by the duration (in seconds) spent learning SHQ controls in the first two levels of the check-point (practice) task that required no spatial memory to solve. In PI levels, participants navigate along a river to find a flare gun, and shoot it back to the starting point by choosing one direction from three alternatives. Performance here was measured as the number of correct answers obtained at the end of all PI levels. Greater number of correct answers indicates better PI performance. The RAM levels are divided into two parts. In Part 1, three of the six arms are blocked, and participants visit the three free arms to collect a star from each of them. In Part 2, the goal of the participants is to visit the three arms that were blocked in Part 1, and collect the remaining three stars from them. Performance on RAM levels was operationalised as the number of reference memory (RM) and spatial working memory (SWM) “errors” made across all the RAM levels. RM errors refer to the number of visits to the arms already visited in Part 1 that needed to be avoided in Part 2. SWM errors refer to the number of visits made to arms in Part 2 that had already been visited during the second part itself. Fewer errors indicate better RAM performance.  2.3.2 Sea Hero Quest Stress Rating  Participants were asked to rate the stress they experienced while playing SHQ levels on a scale ranging from 0 (“*not at all”*) to 10 (“*extremely”*) once they completed gameplay.  2.4 Visuospatial Ability Measures  The visuospatial abilities of participants were measured using three tasks, which are as follows:  2.4.1 Mental Rotation Test ([23](#_ENREF_23))  The MRT Form A consists of 12 stimuli, each of which is a two-dimensional image of a three-dimensional object drawn by a computer. We modified the MRT Form A to include three answer options and only one correct answer per question instead of four answer options and two correct answers, respectively. We also reduced the time limit from 4 minutes to 3 minutes to maintain adequate time pressure. Participants were presented the printed version, and they marked the answer option they thought was the rotated version of the target stimulus. Scores range from 0 to 12. Higher scores indicate better performance.  2.4.2 Design Organization Test ([25](#_ENREF_25))  Participants completed the printed version of both Form A and Form B of the DOT in a counterbalanced manner. At the top of the page, there is a row of six squares that is numbered from 1 to 6, which serves as a code key for completing this task. There are nine square grids below this, each of which showcases a unique pattern composed of a specific combination of the numbered squares in the code key. Participants complete empty grids below these patterned grids using the corresponding numbered squares from the code key. As it is possible to get a ceiling effect within two minutes, participants were allotted one minute to complete this measure ([50](#_ENREF_50)). Scores range from 0 to 112. Higher scores indicate better performance.  2.4.3 Digital Corsi ([31](#_ENREF_31))  In this task, participants observed a set of nine blocks on the computer screen. A number of the observed blocks lit up in a particular sequence, starting with three blocks and increasing with every successful trial. Participants then repeated the sequence by clicking on the blocks in the order in which they lit up. The sum of the number of blocks they clicked in the correct order was computed as their total score, which could range from 0 to 150. Higher scores indicate better performance. It is important to note the digital version of the Corsi task is conceptually different from the regular manual version. Specifically, in the digital version, the absence of predictive finger movements results in slightly different processing and performance.  2.5 Navigation Measures  Information about navigation preferences and strategies of participants was obtained using the measures listed below, which participants completed on a computer.  2.5.1 Santa Barbara Sense of Direction Scale ([38](#_ENREF_38))  The SBSOD was used to assess the perceived sense of direction of participants. The instrument consists of 15 items, which are measured on a seven-point scale (“*strongly agree*” to “*strongly disagree*”). Total scores range from 1 to 7. Higher scores indicate better perceived sense of direction. The SBSOD has been demonstrated to have high internal consistency (α = .88) and test-retest reliability (α = .91), which was assessed by administering the questionnaire 40 days apart. Construct validity was determined by significant correlations between SBSOD scores and wayfinding performance.  2.5.2 Navigation Strategies Questionnaire ([35](#_ENREF_35))  The NSQ consists of 14 items that evaluate the propensity for map-based navigation. The difference between the number of map-based answers and non-map-based answers is taken as a measure of mapping tendency. Total scores range from -14 to +14. Higher scores represent greater use of map-based strategy during navigation.  2.5.3 Radial Arm Maze Navigation Strategy  After the completion of every RAM level, participants were presented with a multiple-choice question, which required them to indicate the type of navigation strategy they used to complete the level. Participants were asked, “*How did you navigate? (1) Counted from the start; (2) Used multiple landmarks; (3) Counted from the landmark*.*”* We recorded the most frequently used navigation strategy. Participants were categorised as having used a landmark strategy if they indicated either of the latter two options on at least three out of five RAM levels. The same was done for counting strategy if the first option was indicated on the majority of RAM levels. If participants skipped a question, and their dominant navigation strategy could not be determined (i.e., they had used counting strategy and landmark strategy on two levels each), their data were excluded from the analysis.  2.6 Procedure  The study was conducted over two sessions, which were held a week apart from each other. Before the first session, participants attempted the MRT to provide an initial measure of their visuospatial ability. In the first experimental session, participants played SHQ wayfinding and PI levels for six minutes each. Before starting SHQ gameplay, they completed practice levels as part of a brief tutorial to learn the controls of the game. At the end of the session, participants indicated their stress level during gameplay, and attempted the DOT. Upon their return a week later, participants played RAM levels, and indicated their navigation strategy for each level. They also performed the D-Corsi, and completed the SBSOD and the NSQ.  2.7 Power Analysis  Based on the estimates from the prior work motivating the approach, the present study assumed the effect size of *d* = .80 ([51](#_ENREF_51)). Using the conversion calculations by Ruscio ([52](#_ENREF_52)), we determined that *d* = .80 amounts to *r* = 0.371. Sample size calculations using these values revealed that a minimum of 72 participants were required to achieve 90% power in order to detect a difference at the 5% significance level.” |
| --- | --- | --- | --- | --- |
| Setting | 5 | Describe the setting, locations, and relevant dates, including periods of recruitment, exposure, follow-up, and data collection | 11, 16 | Methods of recruitment are described under the “2.2 Participants” sub-header of the Methods section. This is as follows:  “Seventy-eight healthy English-speaking participants over the age of 18 years (*M* = 20.33 years, *SD* = 4.03 years) were recruited from University College London through its online subject pool SONA, and compensated in the form of course credits. Demographic information about the participants is summarised in Table 2. Ethical approval was obtained from the University College London Review Board (9807/004). All participants provided informed written consent.”  Information about data collection is included under the “2.6 Procedure” sub-header of the Methods section. This is as follows:  “The study was conducted over two sessions, which were held a week apart from each other. Before the first session, participants attempted the MRT to provide an initial measure of their visuospatial ability. In the first experimental session, participants played SHQ wayfinding and PI levels for six minutes each. Before starting SHQ gameplay, they completed practice levels as part of a brief tutorial to learn the controls of the game. At the end of the session, participants indicated their stress level during gameplay, and attempted the DOT. Upon their return a week later, participants played RAM levels, and indicated their navigation strategy for each level. They also performed the D-Corsi, and completed the SBSOD and the NSQ.” |
| Participants | 6 | (*a*) *Cohort study*—Give the eligibility criteria, and the sources and methods of selection of participants. Describe methods of follow-up  *Case-control study*—Give the eligibility criteria, and the sources and methods of case ascertainment and control selection. Give the rationale for the choice of cases and controls  *Cross-sectional study*—Give the eligibility criteria, and the sources and methods of selection of participants | 10 | Complete information about the eligibility criteria and sources of participant selection is described under the “2.2 Participants” sub-header of the Methods section. This is as follows:  “Seventy-eight healthy English-speaking participants over the age of 18 years (*M* = 20.33 years, *SD* = 4.03 years) were recruited from University College London through its online subject pool SONA, and compensated in the form of course credits. Demographic information about the participants is summarised in Table 2. Ethical approval was obtained from the University College London Review Board (9807/004). All participants provided informed written consent.” |
|  |  | (*b*) *Cohort study*—For matched studies, give matching criteria and number of exposed and unexposed  *Case-control study*—For matched studies, give matching criteria and the number of controls per case | N/A | N/A |
| Variables | 7 | Clearly define all outcomes, exposures, predictors, potential confounders, and effect modifiers. Give diagnostic criteria, if applicable | 11-16 | All the variables related to Sea Hero Quest, visuospatial ability and navigation strategy investigated and analysed in this study have been defined and elaborated upon under various sub-headers in the Methods section.  Information about all the variables (tasks) related to Sea Hero Quest has been included under the “2.3 Sea Hero Quest Tasks” sub-header of the Methods section. This is as follows:  “2.3 Sea Hero Quest Tasks  2.3.1 Sea Hero Quest App ([7](#_ENREF_7), [8](#_ENREF_8), [45](#_ENREF_45))  SHQ is a VR navigation game for mobile and tablet devices in which participants are required to navigate a three-dimensional environment of water bodies. Navigation abilities of participants are assessed through three types of levels: Wayfinding, PI and RAM (see Fig 1). To avoid a ceiling effect, participants played the somewhat “difficult” levels of SHQ. The difficulty of wayfinding levels was based on the number of goals in a particular level, and how far apart they were located from each other. Levels in which there were four or more goals that were located at a considerable distance from each other were considered difficult. For PI levels, difficulty was determined by the number of turns participants had to take to get from the starting point to the final destination. Levels in which there were at least four turns were selected. Based on the aforementioned criteria, we chose levels 16, 37, 32 and 43 for wayfinding to keep a broader selection of levels in terms of spatial characteristics of the environment. For PI, we selected levels 44, 49, 54, 59, 64, 69 and 74 that were later in the game and more challenging. For the purpose of this study, we only analysed levels that were played by all participants in the original trauma study. Accordingly, we considered levels 16 and 37 for wayfinding, and levels 44, 49, 54 and 74 for PI. Lastly, participants played all the RAM levels as there are only five such levels in SHQ.  In wayfinding levels, participants are required to navigate to checkpoints in an ordered manner based on a map presented to them before the game begins. Performance on wayfinding levels was operationalised as the average inefficiency across all the levels. Lower inefficiency values indicate better wayfinding performance, meaning that participants covered less distance to complete all the levels. To control for prior video gaming experience, we standardised wayfinding inefficiency by dividing it by the duration (in seconds) spent learning SHQ controls in the first two levels of the check-point (practice) task that required no spatial memory to solve. In PI levels, participants navigate along a river to find a flare gun, and shoot it back to the starting point by choosing one direction from three alternatives. Performance here was measured as the number of correct answers obtained at the end of all PI levels. Greater number of correct answers indicates better PI performance. The RAM levels are divided into two parts. In Part 1, three of the six arms are blocked, and participants visit the three free arms to collect a star from each of them. In Part 2, the goal of the participants is to visit the three arms that were blocked in Part 1, and collect the remaining three stars from them. Performance on RAM levels was operationalised as the number of reference memory (RM) and spatial working memory (SWM) “errors” made across all the RAM levels. RM errors refer to the number of visits to the arms already visited in Part 1 that needed to be avoided in Part 2. SWM errors refer to the number of visits made to arms in Part 2 that had already been visited during the second part itself. Fewer errors indicate better RAM performance.  2.3.2 Sea Hero Quest Stress Rating  Participants were asked to rate the stress they experienced while playing SHQ levels on a scale ranging from 0 (“*not at all”*) to 10 (“*extremely”*) once they completed gameplay.”  Information about all the measures of visuospatial ability has been included under the “2.4 Visuospatial Ability Measures” sub-header of the Methods section. This is as follows:  “2 The visuospatial abilities of participants were measured using three tasks, which are as follows:  2.4.1 Mental Rotation Test ([23](#_ENREF_23))  The MRT Form A consists of 12 stimuli, each of which is a two-dimensional image of a three-dimensional object drawn by a computer. We modified the MRT Form A to include three answer options and only one correct answer per question instead of four answer options and two correct answers, respectively. We also reduced the time limit from 4 minutes to 3 minutes to maintain adequate time pressure. Participants were presented the printed version, and they marked the answer option they thought was the rotated version of the target stimulus. Scores range from 0 to 12. Higher scores indicate better performance.  2.4.2 Design Organization Test ([25](#_ENREF_25))  Participants completed the printed version of both Form A and Form B of the DOT in a counterbalanced manner. At the top of the page, there is a row of six squares that is numbered from 1 to 6, which serves as a code key for completing this task. There are nine square grids below this, each of which showcases a unique pattern composed of a specific combination of the numbered squares in the code key. Participants complete empty grids below these patterned grids using the corresponding numbered squares from the code key. As it is possible to get a ceiling effect within two minutes, participants were allotted one minute to complete this measure ([50](#_ENREF_50)). Scores range from 0 to 112. Higher scores indicate better performance.  2.4.3 Digital Corsi ([31](#_ENREF_31))  In this task, participants observed a set of nine blocks on the computer screen. A number of the observed blocks lit up in a particular sequence, starting with three blocks and increasing with every successful trial. Participants then repeated the sequence by clicking on the blocks in the order in which they lit up. The sum of the number of blocks they clicked in the correct order was computed as their total score, which could range from 0 to 150. Higher scores indicate better performance. It is important to note the digital version of the Corsi task is conceptually different from the regular manual version. Specifically, in the digital version, the absence of predictive finger movements results in slightly different processing and performance.”  Information about all the measures of visuospatial ability has been included under the “2.5 Navigation Measures” sub-header of the Methods section. This is as follows:  “Information about navigation preferences and strategies of participants was obtained using the measures listed below, which participants completed on a computer.  2.5.1 Santa Barbara Sense of Direction Scale ([38](#_ENREF_38))  The SBSOD was used to assess the perceived sense of direction of participants. The instrument consists of 15 items, which are measured on a seven-point scale (“*strongly agree*” to “*strongly disagree*”). Total scores range from 1 to 7. Higher scores indicate better perceived sense of direction. The SBSOD has been demonstrated to have high internal consistency (α = .88) and test-retest reliability (α = .91), which was assessed by administering the questionnaire 40 days apart. Construct validity was determined by significant correlations between SBSOD scores and wayfinding performance.  2.5.2 Navigation Strategies Questionnaire ([35](#_ENREF_35))  The NSQ consists of 14 items that evaluate the propensity for map-based navigation. The difference between the number of map-based answers and non-map-based answers is taken as a measure of mapping tendency. Total scores range from -14 to +14. Higher scores represent greater use of map-based strategy during navigation.  2.5.3 Radial Arm Maze Navigation Strategy  After the completion of every RAM level, participants were presented with a multiple-choice question, which required them to indicate the type of navigation strategy they used to complete the level. Participants were asked, “*How did you navigate? (1) Counted from the start; (2) Used multiple landmarks; (3) Counted from the landmark*.*”* We recorded the most frequently used navigation strategy. Participants were categorised as having used a landmark strategy if they indicated either of the latter two options on at least three out of five RAM levels. The same was done for counting strategy if the first option was indicated on the majority of RAM levels. If participants skipped a question, and their dominant navigation strategy could not be determined (i.e., they had used counting strategy and landmark strategy on two levels each), their data were excluded from the analysis.” |
| Data sources/ measurement | 8* | For each variable of interest, give sources of data and details of methods of assessment (measurement). Describe comparability of assessment methods if there is more than one group | 11-16 | Same as item 7 |
| Bias | 9 | Describe any efforts to address potential sources of bias | N/A | N/A |
| Study size | 10 | Explain how the study size was arrived at | 16 | The sample size of the study was determined by a power analysis, which is detailed under the “2.7 Power Analysis”. This is as follows:  “2.7 Power Analysis  Based on the estimates from the prior work motivating the approach, the present study assumed the effect size of *d* = .80 ([51](#_ENREF_51)). Using the conversion calculations by Ruscio ([52](#_ENREF_52)), we determined that *d* = .80 amounts to *r* = 0.371. Sample size calculations using these values revealed that a minimum of 72 participants were required to achieve 90% power in order to detect a difference at the 5% significance level.” |
| Quantitative variables | 11 | Explain how quantitative variables were handled in the analyses. If applicable, describe which groupings were chosen and why | 11-16 | Same as item 7 |
| Statistical methods | 12 | (*a*) Describe all statistical methods, including those used to control for confounding | 17 | All the statistical methods are described under the “2.8 Data Analysis” sub-header in the Methods section. This is as follows:  “The data were analysed using IBM SPSS Statistics (Version 28) ([53](#_ENREF_53)). Extreme outliers were excluded from the analysis of the task in question, though their data for other tasks were retained if they were not outliers on them. Pearson correlation analysis was used to explore the relationship between SHQ and all the measures of visuospatial abilities, navigation and stress. Correlations for predicted relationships were evaluated at the *p* < .05 threshold (see Table 1), while all the other correlational analyses were Bonferroni-corrected for multiple comparisons, *p* <.0009 (55 comparisons) to control for Type I error. Independent samples *t*-tests were also performed to examine how performance between those who used landmark strategy or counting strategy most frequently on RAM levels differ on other measures. Finally, Pearson chi-square test of independence was used to examine the relationship between the propensity for map-based navigation (as indicated by the NSQ) and the type of navigation strategy used on RAM levels (used landmark strategy or counting strategy).” |
|  |  | (*b*) Describe any methods used to examine subgroups and interactions | 17 | This is described in item 12. Interaction examination was not applicable. |
|  |  | (*c*) Explain how missing data were addressed | 17 | This is described under the “2.8 Data Analysis” sub-header in the Methods section. This is as follows:  “Missing values were not inferred.” |
|  |  | (*d*) *Cohort study*—If applicable, explain how loss to follow-up was addressed  *Case-control study*—If applicable, explain how matching of cases and controls was addressed  *Cross-sectional study*—If applicable, describe analytical methods taking account of sampling strategy | N/A | N/A |
|  |  | (*e*) Describe any sensitivity analyses | N/A | N/A |
| **Results** |  |  |  |  |
| Participants | 13* | (a) Report numbers of individuals at each stage of study—eg numbers potentially eligible, examined for eligibility, confirmed eligible, included in the study, completing follow-up, and analysed | 11 | This is described under the “2.2. Participants” sub-header of the Methods section,  “Seventy-eight healthy English-speaking participants over the age of 18 years (*M* = 20.33 years, *SD* = 4.03 years) were recruited from University College London through its online subject pool SONA, and compensated in the form of course credits. Demographic information about the participants is summarised in Table 2. Ethical approval was obtained from the University College London Review Board (9807/004). All participants provided informed written consent.” |
|  |  | (b) Give reasons for non-participation at each stage | N/A | N/A |
|  |  | (c) Consider use of a flow diagram | N/A | The use of a flow diagram was not deemed appropriate. |
| Descriptive data | 14* | (a) Give characteristics of study participants (eg demographic, clinical, social) and information on exposures and potential confounders | 11, 17-18 | Demographic details of the participants are summarised in Table 2 under the “2.2. Participants” sub-header of the Methods section, whereas the details of their performance on across all the variables are summarised in Table 3 in the Results section. “Table 2: Demographics overview for participants in the study.”  “Table 3: Descriptive statistics of participant performance across all measures.” |
|  |  | (b) Indicate number of participants with missing data for each variable of interest | 16-17 | The total numbers of participants recorded for each variable are stated in Table 3 of the Results section.  “Table 3: Descriptive statistics of participant performance across all measures.” |
|  |  | (c) *Cohort study*—Summarise follow-up time (eg, average and total amount) | N/A | N/A |
| Outcome data | 15* | *Cohort study*—Report numbers of outcome events or summary measures over time | N/A | N/A |
|  |  | *Case-control study—*Report numbers in each exposure category, or summary measures of exposure | N/A | N/A |
|  |  | *Cross-sectional study—*Report numbers of outcome events or summary measures | 8, 11-16 | Same as items 3 and 7 |
| Main results | 16 | (*a*) Give unadjusted estimates and, if applicable, confounder-adjusted estimates and their precision (eg, 95% confidence interval). Make clear which confounders were adjusted for and why they were included | N/A | We report unadjusted estimates in the Results and the Appendix sections. |
|  |  | (*b*) Report category boundaries when continuous variables were categorized | 11 | Category boundaries have been set for only one continuous variable which is the age variable, and have been displayed in Table 2 under the “2.2 Participants” sub-header in the Methods section.  “Table 2: Demographics overview for participants in the study.” |
|  |  | (*c*) If relevant, consider translating estimates of relative risk into absolute risk for a meaningful time period | N/A | N/A |
| Other analyses | 17 | Report other analyses done—eg analyses of subgroups and interactions, and sensitivity analyses | N/A | N/A |
| **Discussion** |  |  |  |  |
| Key results | 18 | Summarise key results with reference to study objectives | 26 | This is done in the first paragraph of the Discussion section, which is follows:  “We tested participants with virtual navigation tasks (wayfinding, PI and RAM) in the gaming app SHQ, visuospatial abilities (mental rotation, visuospatial processing and VSWM), and navigation strategies and preferences (sense of direction, mapping tendency and RAM navigation strategy) to better understand how these cognitive constructs relate to each other. The different constructs showed low levels of association, with negligible correlation among the three spatial navigation tasks on SHQ, and weak correlation between these and the self-ratings and navigation strategies. We observed modest correlations between each of the three visuospatial abilities, and all of them with wayfinding, but not with other navigation tasks. We discuss what these results mean for understanding cognitive profiles of navigation ability.” |
| Limitations | 19 | Discuss limitations of the study, taking into account sources of potential bias or imprecision. Discuss both direction and magnitude of any potential bias | 29-30 | This is done in the eighth paragraph of the Discussion section, which is follows:  “There are a number of limitations to our study that should be considered. First, our sample size was limited by the challenge of conducting in lab testing, which was central to our planned design. Relatedly, the sample was overwhelmingly composed of young and female participants, which could impact the external validity of the findings. Since previous research has shown that differences based on sex, age and culture exist in navigation performance and related behaviours, it would be helpful to use large samples to explore a broader range of ages, and compare gender and performance across nations in the future ([7](#_ENREF_7)). Nonetheless, our results elucidate the relationship among object-based spatial ability, navigation strategy and virtual navigation performance specifically for young women. Second, the visuospatial tasks we used here were adapted from clinically used tests aimed at detecting differences between groups, whereas our aim was to explore variation in the population. Thus, it may be useful to further develop such tests to help optimally explore individual differences ([70](#_ENREF_70)). Finally, as with all correlative approaches to assessing individual differences, the capacity of the tests used will depend partly on the variance with the data generated by the test in the group tested. In our current study, the PI task had less variability than others, and thus, this may have impacted our capacity to detect some relationship between PI and other measures. Nonetheless, we were able to observe predicted correlation between this test and the D-Corsi, indicating the variation present was still sufficient to detect some effects.” |
| Interpretation | 20 | Give a cautious overall interpretation of results considering objectives, limitations, multiplicity of analyses, results from similar studies, and other relevant evidence | 26-30 | This has been done throughout Discussion, particularly in paragraph 8 in this section. These aforementioned paragraphs are as follows:  “  Consistent with our predictions, we found a significant correlation between wayfinding performance and performance on visuospatial tasks of mental rotation, visuospatial processing and VSWM. To our knowledge, this is the first study to explore the relationship between visuospatial processing as measured by the DOT and navigation tasks. In all three cases, a low-to-moderate correlation is in line with previous studies examining the relationship between large-scale and small-scale spatial abilities ([18](#_ENREF_18), [21](#_ENREF_21)). The absence of strong correlations between these abilities indicates that while wayfinding and these constructs may have some overlap in the cognition required, they also have different demands. This is a pattern discussed in past research exploring the relation between small-scale spatial abilities and large-scale wayfinding ability using SHQ and real-world navigation tasks involving various measures such as accuracy, reaction time, distance travelled and number of errors ([11](#_ENREF_11)). These findings are useful in highlighting the potential utility of VR-based navigation tasks in capturing something that is distinct from object-based visuospatial tasks. One factor that may differentiate wayfinding from the other visuospatial tasks is the reliance on broader executive functions demands. Unlike object-based visuospatial tasks, wayfinding places specific demands on planning and inhibition ([54](#_ENREF_54), [55](#_ENREF_55)). In SHQ, this involves avoiding re-approaching visited checkpoints and planning optimal paths given the order of checkpoints indicated on the initially shown map.  Interestingly, the three tests of visuospatial ability have a moderate correlation with each other, indicating that they measure different but related cognitive aspects. Mental rotation may require flexible switching between motor simulation and analytic thinking, depending on the difficulty level ([56](#_ENREF_56)). Block tapping may involve control processes, visual working memory and visuospatial attention ([57](#_ENREF_57), [58](#_ENREF_58)). Visuospatial processing on the DOT may rely on both visuospatial abilities and problem-solving skills ([59](#_ENREF_59)). This may explain why visuospatial processing on the DOT has the strongest correlation with wayfinding performance, considering the link between wayfinding performance and executive functions.  We had predicted that performance on RAM levels and VSWM as measured by D-Corsi performance would be correlated because both require holding a set of locations in mind over many seconds i.e., they test spatial working memory. We found no evidence for this. This result is consistent with the view that ‘spatial working memory’ is not a unitary cognitive function, but depends on the context (e.g., 2D screen space versus VR-rendered 3D environment). Theoretically, it seems plausible that the D-Corsi is supported mainly via frontoparietal circuits, and that the RAM additionally draws on hippocampal circuits to support the representation of the large-scale environment ([45](#_ENREF_45), [60](#_ENREF_60)). The absence of a correlation between the RAM and other navigation tasks further indicates that different cognitive demands between the tasks are involved.  A surprising result was that neither sense of direction nor mapping tendency was correlated with navigation performance on any SHQ task. This stands in contrast with previous evidence of correlations between navigation behaviour and these constructs ([35](#_ENREF_35), [38](#_ENREF_38), [61](#_ENREF_61)), but is similar to some other evidence ([62](#_ENREF_62)). It may be that participants need to physically move or use body-based cues to navigate in a space for a stronger association of navigation behaviours with standardised measures such as the SBSOD (e.g., Hegarty et al. ([18](#_ENREF_18))). Another possibility is that people tend to rate their navigation ability in relation to how often they might get lost. It is likely that participants use GPS-based systems to find their way, and might rarely find themselves in the situation simulated in the wayfinding task where a map is studied and must be committed to memory before navigation. When we recently sampled a large population, drawn from many nations and a range of ages, we found a consistent relationship between wayfinding performance in SHQ and self-rated navigation ability ([9](#_ENREF_9)). Thus, the relationship between self-ratings and wayfinding performance is likely moderated by a wide range of factors (e.g., He & Hegarty ([63](#_ENREF_63)), Hegarty et al. ([18](#_ENREF_18)), van der Ham & Koutzmpi ([64](#_ENREF_64)), van der Ham et al. ([65](#_ENREF_65))). Notably, while actual performance in the wayfinding task in SHQ was not correlated with sense of direction or mapping tendency, the stress ratings of playing SHQ were, even though they did not reach the stringent Bonferroni-corrected significance level. Thus, people who think they are good navigators, or those who tend to think using maps, might be less likely to find SHQ stressful. In future, it will be useful to explore more about spatial anxiety in daily life and SHQ performance to understand if they are linked more than the stress reported ([44](#_ENREF_44), [66](#_ENREF_66)). It is worth mentioning that the baseline stress and mood ratings of participants could not be recorded due to a technical error. Hence, it is possible that SHQ stress ratings were not an accurate measure of the stress caused by the navigation tasks; the stress may have simply been a result of being in a laboratory setting, or a prior event before the assessment.  We found that the SBSOD and the NSQ are moderately correlated with each other, which indicates that having a strong sense of direction is associated with using map-based strategy to navigate. To the authors’ knowledge, the association between these two constructs has been explored for the first time. This finding indicates that the two tests capture related dimensions of navigational profiles and attitudes, but not so overlapping as to be equivalent.  We found that those who used the landmark strategy on RAM levels performed significantly better on both RAM and wayfinding levels than those who used the counting strategy. This mirrors recent evidence from the large participant group of over 37,000 participants ([45](#_ENREF_45)). By combining the NSQ with the RAM tests for this study we reveal, for this population, that landmark-counting strategies appear to be orthogonal to the survey-route dimension captured by the NSQ. Notably, the lack of a correlation between wayfinding inefficiency and the RAM errors in this small lab sample matches our recent report of an absence of correlation between these in an online sample of over 37,000 participants ([45](#_ENREF_45)). The absence of a correlation of the PI measure and the other navigation tasks is notable in light of recent evidence that PI, rather than other spatial tests, might be particularly important for early detection of AD ([67-69](#_ENREF_67)).  There are a number of limitations to our study that should be considered. First, our sample size was limited by the challenge of conducting in lab testing, which was central to our planned design. Relatedly, the sample was overwhelmingly composed of young and female participants, which could impact the external validity of the findings. Since previous research has shown that differences based on sex, age and culture exist in navigation performance and related behaviours, it would be helpful to use large samples to explore a broader range of ages, and compare gender and performance across nations in the future ([7](#_ENREF_7)). Nonetheless, our results elucidate the relationship among object-based spatial ability, navigation strategy and virtual navigation performance specifically for young women. Second, the visuospatial tasks we used here were adapted from clinically used tests aimed at detecting differences between groups, whereas our aim was to explore variation in the population. Thus, it may be useful to further develop such tests to help optimally explore individual differences ([70](#_ENREF_70)). Finally, as with all correlative approaches to assessing individual differences, the capacity of the tests used will depend partly on the variance with the data generated by the test in the group tested. In our current study, the PI task had less variability than others, and thus, this may have impacted our capacity to detect some relationship between PI and other measures. Nonetheless, we were able to observe predicted correlation between this test and the D-Corsi, indicating the variation present was still sufficient to detect some effects.  In conclusion, our study highlights that many tests and self-rating scales for navigation and visuospatial abilities can be highly non-overlapping, at least in a UK university student sample. Our results help further characterise what the different tests used in SHQ are related to. For example, we show that wayfinding has some overlap with object-based visuospatial tasks, whereas PI and RAM tasks have much less. The findings also point to the limitations of standardised tests of spatial cognition in capturing the nuances of navigation performance, particularly the distinction between small- and large-scale functioning. In future research it would be useful to probe a broader range of environments to understand how the complexity of the layouts (12) and landmark density (71, 72) lead to higher or lower correlations with object-based visuospatial ability.” |
| Generalisability | 21 | Discuss the generalisability (external validity) of the study results | 29 | We note that the generalizability of our results in paragraph 8 of the Discussion section:  “Relatedly, the sample was overwhelmingly composed of young and female participants, which could impact the external validity of the findings. Since previous research has shown that differences based on sex, age and culture exist in navigation performance and related behaviours, it would be helpful to use large samples to explore a broader range of ages, and compare gender and performance across nations in the future ([7](#_ENREF_7)). Nonetheless, our results elucidate the relationship among object-based spatial ability, navigation strategy and virtual navigation performance specifically for young women.” |
| **Other information** |  |  |  |  |
| Funding | 22 | Give the source of funding and the role of the funders for the present study and, if applicable, for the original study on which the present article is based |  | This information is included under the “Funding” header.  “This research was supported by funding by Alzheimer’s Research UK for Sea Hero Quest (Grant: ARUK-DT2016-1).” |

*Give information separately for cases and controls in case-control studies and, if applicable, for exposed and unexposed groups in cohort and cross-sectional studies.

**Note:** An Explanation and Elaboration article discusses each checklist item and gives methodological background and published examples of transparent reporting. The STROBE checklist is best used in conjunction with this article (freely available on the Web sites of PLoS Medicine at http://www.plosmedicine.org/, Annals of Internal Medicine at http://www.annals.org/, and Epidemiology at http://www.epidem.com/). Information on the STROBE Initiative is available at www.strobe-statement.org.
