# Supplementary material for: The relationship between object-based spatial ability and virtual navigation performance
Source: PLoS One. 2024 May 9;19(5):e0298116. doi: 10.1371/journal.pone.0298116 (PMC11081363; doi:10.1371/journal.pone.0298116)
Supplement: S1 Appendix — (DOCX) [file pone.0298116.s002.docx]

**Appendix**

**Gender-related Analysis**

Though this paper was not specifically designed to measure gender differences, we explored gender-specific responses as part of a thorough investigation of the dataset. Gender-related analysis revealed significant differences in performances with respect to RAM errors and NSQ score (see Appendix Table 1). Male participants made significantly fewer RM and SWM errors on RAM levels than female participants. Further, male participants had a significantly stronger mapping tendency as demonstrated by the NSQ. There was no association between gender and the type of strategy used to navigate RAM levels (*p* > .05).

Appendix Table 1

Metrics found to be significantly different for male participants versus female participants across measures (one-tailed).

| Measure | Female | | Male | | Statistics | | | |
| --- | --- | --- | --- | --- | --- | --- | --- | --- |
|  | *M* | *SD* | *M* | *SD* | *t* | *df* | *p* | *d* |
| RAM RM Errors | 5.34 | 2.52 | 4.08 | 2.61 | 1.91 | 75 | 0.029 | 0.499 |
| RAM SWM Errors | 1.07 | 1.19 | 0.58 | 0.85 | 1.71 | 75 | 0.045 | 0.445 |
| SHQ Tutorial Duration | 47.36 | 6.81 | 44.08 | 4.61 | 1.94 | 72 | 0.028 | 0.519 |
| NSQ Score | -2.44 | 4.80 | -0.10 | 5.38 | -1.81 | 75 | 0.037 | -0.472 |
| SHQ Stress Rating* | 4.44 | 2.39 | 2.60 | 1.50 | 3.98 | 53.49 | <0.001 | 0.836 |

*Note.* Non-significant metrics are not reported. * = Statistics reported for SHQ stress ratings are not assuming equal variances as the Levene’s Test was significant (*F* = 6.683, *p* = 0.012).

**Wayfinding and Path Integration Performance**

We investigated the correlation between wayfinding and PI performance in the large Sea Hero Quest dataset (Coutrot et al., 2022a). We wanted to quantify how this correlation varies across training, wayfinding and path integration levels of different difficulties. We selected the participants who completed all Sea Hero Quest levels and were between 19 and 70 years old (N = 12,111, 5,979 males, mean age = 40.08, SD = 15.15 years). First, we computed the correlation between the overall PI performance (sum of correct answers across all PI levels) and the trajectory length of each WF level (see Fig 3). As expected, we found a small correlation for training levels 1 and 2 (*r* =-0.07) and a stable moderate correlation for all wayfinding levels (*r* ~ -0.3). Then, we computed the point-biserial correlation between the overall training and wayfinding performance (the 1st component of a PCA across the trajectory lengths of the training levels on one side, and of the wayfinding levels on the other side) and the Boolean vector of correct answers for each PI levels (see Fig 4). As expected, we found a small correlation between training and PI performance and a stable moderate correlation between wayfinding and PI performance for the first 10 PI levels (until level 49). For the next PI levels, the magnitude of the correlation unexpectedly decreases to reach the same level as with the training performance.

*
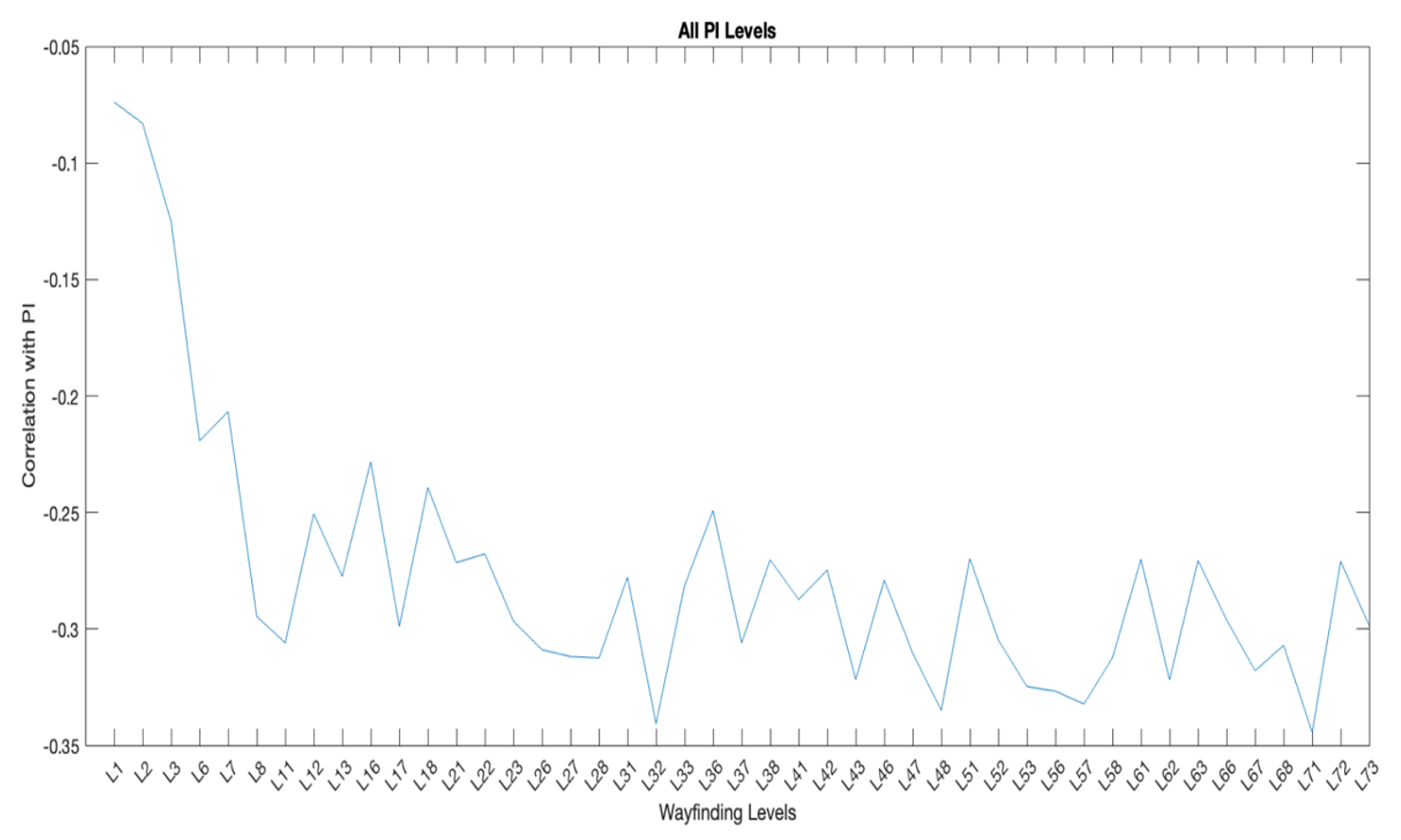
*

**Fig 3. Correlation between the overall Path Integration performance and the trajectory lengths of each Wayfinding level.**

**
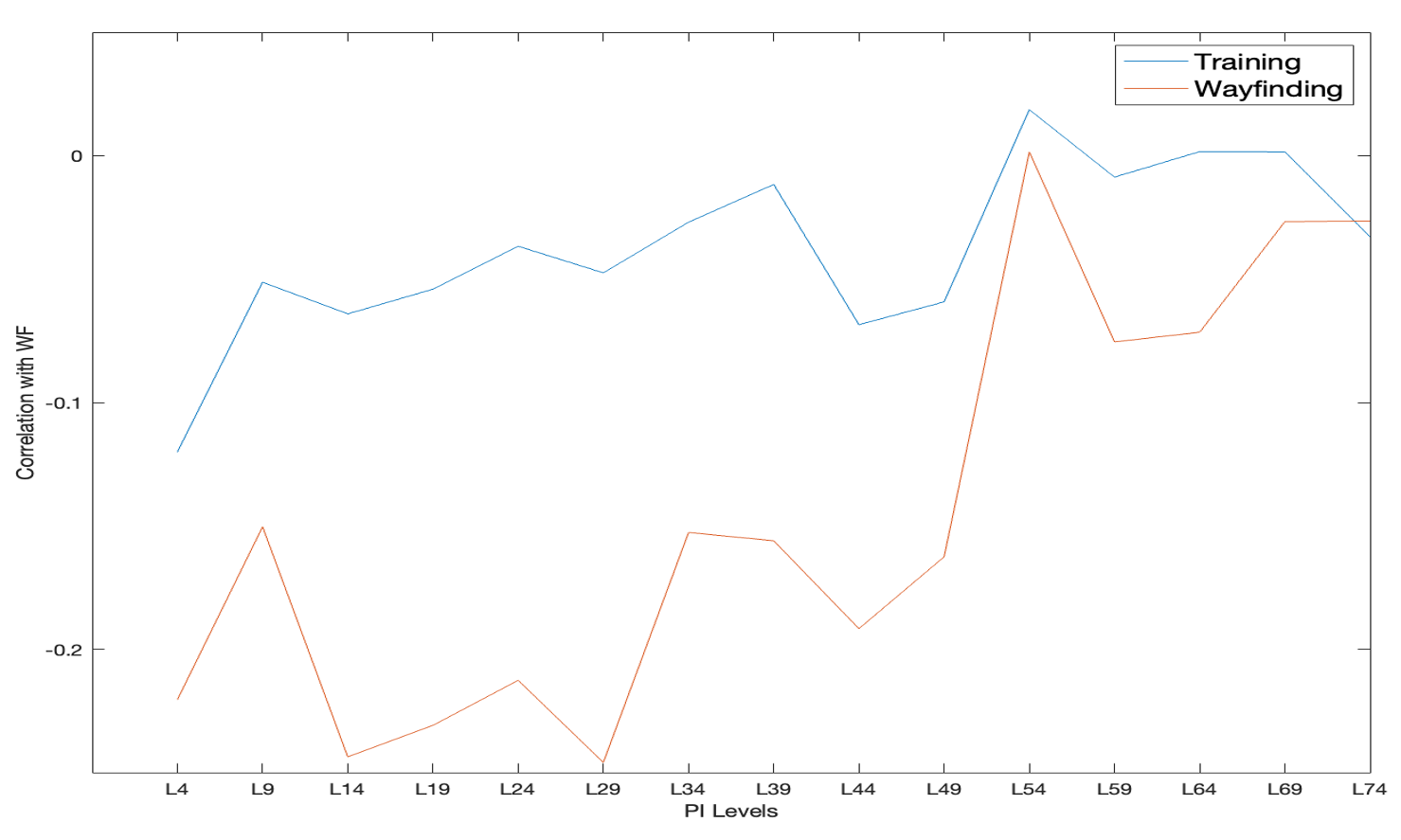
**

**Fig 4. Point-biserial correlation between the overall Training and Wayfinding performance and the Boolean answers for each Path Integration level.**
