# Supplementary material for: The relationship between object-based spatial ability and virtual navigation performance
Source: PLoS One. 2024 May 9;19(5):e0298116. doi: 10.1371/journal.pone.0298116 (PMC11081363; doi:10.1371/journal.pone.0298116)
Supplement: Attachment — Submitted filename: ResponseToReviewers.docx [file pone.0298116.s004.docx]

**REPLY TO REVIEWS:**

We are grateful to have the opportunity to reply to the reviews and re-submit.

Below we quote comments from the Editor and Reviewers in ***italics***, our response in **blue** font and quotes from our updated manuscript in **red** font (as in the red font in the re-submitted manuscript).

**EDITOR COMMENTS:**

*I would strongly urge the authors to give careful consideration to the reasoning, discussion, and conclusion presented in the manuscript, as well as the examination of the sample and its comparison to data from other articles published on SHQ and relevant literature. Extensive revisions and possible restructuring will be required, particularly in the Discussion and Conclusion sections of the document.*

Thank you for encouraging us to carefully consider these particular elements. We have substantially revised the manuscript in light of the reviews.

**REVIEWER 1 COMMENTS:**

**Overall:**

*In their study “The relationship between object-based spatial ability and virtual navigation performance“, Garg et al. compare the performance in a mobile gaming app from 78 participants with established questionnaires or other tests for visuospatial abilities (mental rotation, visuospatial working memory, visuospatial processing, self-assessment of orientation). Surprisingly, only sparse strong correlations were found. The authors explain this finding by different subqualities being assessed in the individual tests.*

*The manuscript is overall well written and the methodology is sound. The mobile gaming app is well-established and its application in comparison with other test batteries can offer valuable insight into spatial abilities.*

*However, my main concern is the small sample size, consisting of young, predominantly female participants. This factor needs further emphasis when discussing the results.*

We are grateful for the suggestion to discuss these issues further. They are indeed an important limitation of the study, and we believe adding discussion of them has strengthened the depth of the manuscript. Please refer to L567-573 in the discussion section.

“Relatedly, the sample was overwhelmingly composed of young and female participants, which could impact the external validity of the findings. Since previous research has shown that differences based on sex, age and culture exist in navigation performance and related behaviours, it would be helpful to use large samples to explore a broader range of ages, and compare gender and performance across nations in the future (7). Nonetheless, our results elucidate the relationship among object-based spatial ability, navigation strategy and virtual navigation performance specifically for young women.”

*One further aspect that would require major revision is the analysis of stress level during testing which is not compared to baseline stress or anxiety levels.*

Thank you for highlighting this. Unfortunately, due to a technical error at the time of data collection, the baseline stress and mood ratings of participants could not be recorded. We have now clarified as such in the discussion section, and have also discussed the implications of the absence of baseline stress levels. Please refer to L542-546 in the discussion section.

“It is worth mentioning that the baseline stress and mood ratings of participants could not be recorded due to a technical error. Hence, it is possible that SHQ stress ratings were not an accurate measure of the stress caused by the navigation tasks; the stress may have simply been a result of being in a laboratory setting, or a prior event before the assessment.”

*The manuscript fulfills the PLOS ONE publication criteria and, apart from the aforementioned concern, only requires minor revisions.*

Thank you for supporting minor revisions.

**Abstract:**
*1. Please provide information on the participants (N=78, 20 male, mean age 20.33yrs).*

Thank you for pointing this out. We have now mentioned participant information in L68-L71 in the abstract section.

“Here, we report the use of an established mobile gaming app, Sea Hero Quest (SHQ), as a measure of navigation ability in a sample of young, predominantly female university students (N = 78; 20; female = 74.3%; mean age = 20.33 years).”

*2. Line 66 “disease monitoring in Alzheimer’s Disease“: given how spatial impairment can occur in other neurological disorders outside of AD, consider rephrasing the sentence. A clinically applicable test for spatial impairment would benefit not only dementia-patients but other fields of neurology, too. (again, in Introduction, line 95). E.g., Borel, L., et al. "Vestibular syndrome: a change in internal spatial representation." Neurophysiologie Clinique/Clinical Neurophysiology 38.6 (2008): 375-389., Mitchell, Peter, and Danielle Ropar. "Visuospatial abilities in autism: A review." Infant and Child Development: An International Journal of Research and Practice 13.3 (2004): 185-198.,* ***…***

Thank you for suggesting this. We have added multiple sclerosis, vestibular syndromes and autism as three more examples of disorders in L91-93 in the introduction section.

“Deficits in these competencies may constitute an early marker of conditions such as Alzheimer’s disease (AD), multiple sclerosis, vestibular syndrome and autism (1-4).”

**Introduction:**
*1. Line 95: “There are also negative effects of disorientation and its associated risks“: very vague, please explain what you mean by this****.***

Thank you for raising this point. We have rephrased L93-94 in the introduction section to make our point clearer.

“Furthermore, navigation impairments like disorientation can affect one’s quality of life by causing distress and impediments in daily functioning (5, 6).”

*2. Line 109: “…can be partly predicted by gender inequality“ and line 111, “…peaks at seven hours of sleep…“: consider rephrasing these sentences, since right now the meaning remains unclear.*

Thank you for letting us know. We have rephrased L112-116 to make this information easier to understand.

“Studies using SHQ have revealed that gender differences in navigation ability for a country can be partially attributed to gender inequality (8). They have also found that individuals are more adept at navigating environments that are topologically similar to those in which they were raised (12). Such studies have also shown that performance is best for older participants who report sleeping 7 hours per night (13).”

*3. The hypotheses in table 1 should rather be part of the introduction then their own table; in the current form, the table doesn’t add further value for the reader.*

Thank you for suggesting this. We have added another paragraph at the end of the introduction section (L171-188) to make the predictions in Table 1 part of the text. However, we have also retained Table 1 as we feel it complements the newly added paragraph and makes it easier to visualise all of the 20 predictions in our study.

“In this study, we explore how visuospatial abilities, navigation strategy and gameplay stress relate to performance on SHQ and to each other. Based on the literature (e.g., Wolbers & Hegarty (17)), we made 20 predictions. We expected wayfinding to correlate with other measures due to its diverse demands such as perception, memory, decision-making, etc. Specifically, we predicted that longer duration to complete wayfinding levels (i.e., wayfinding inefficiency) would be associated with lower mental rotation, visuospatial processing, VSWM, sense of direction and mapping tendency as measured by the MRT, DOT, D-Corsi, SBSOD and NSQ, respectively. Greater wayfinding inefficiency would also be related to higher total SHQ gameplay duration and SHQ stress. We further predicted that more correct answers on PI levels would be associated with stronger VSWM and sense of direction as measured by D-Corsi and SBSOD, respectively. Moreover, we hypothesized that the number of reference memory errors (i.e., RM errors) and spatial working memory errors (i.e., SWM errors) on RAM levels would positively correlate with each other and negatively with D-Corsi and SBSOD scores. Additionally, we predicted that mental rotation and visuospatial processing, as measured by the MRT and the DOT, respectively, would positively correlate with each other and with VSWM, as measured by the D-Corsi. Our final hypothesis was that sense of direction and mapping tendency, as measured by the SBSOD and the NSQ, respectively, would positively correlate with each other. Table 1 below shows the 20 hypothesised relationships between performance on different tasks.”

*4. Line 118: please indicate that a predominantly female, young cohort was tested.*

Thank you for raising this. We have now edited the manuscript to specifically mention this in L68-71 in the abstract, L160-162 in the introduction and L542-546 (please see our first response under the “overall” sub-header for Reviewer 1 Comments) in the discussion section.

L68-71: “Here, we report the use of an established mobile gaming app, Sea Hero Quest (SHQ), as a measure of navigation ability in a sample of young, predominantly female university students (N = 78; 20; female = 74.3%; mean age = 20.33 years).”

L160-162: “We test the navigation abilities of a predominantly female sample of university students on three tasks in SHQ: wayfinding, path integration (PI), and the radial arm maze (RAM) test of spatial memory.”

**Methods:**
*1. Line 153: „no psychological disorder“: were any neurological disorders evident? Did any of the participants have a history of neurological diseases potentially affecting visuospatial abilities?*

Participants were only asked about a history of psychological disorders. Nonetheless, we did not observe any evident neurological disorders, and participants did not report any difficulties preventing them from performing the tasks well.

*2. Methods 2.4.1, line 219: how were the images presented? Printed, on screen, …?*

Thank you for pointing this out. We have now specified the modality of test administration for all the questionnaires and tasks used in the study. Please see pages 14-15.

L282-283: “Participants were presented the printed version, and they marked the answer option they thought was the rotated version of the target stimulus.”

L287-288: “Participants completed the printed version of both Form A and Form B of the DOT in a counterbalanced manner.”

L305-306: “Information about navigation preferences and strategies of participants was obtained using the measures listed below, which participants completed on a computer.”

*3. Methods 2.4.2: from the description alone, I had trouble understanding the DOT. Consider rephrasing the paragraph to make it clearer.*

Thank you for flagging this to us. We have rephrased the paragraph about DOT to make it easier to understand. Please see L287-294 in the methodology section.

“Participants completed the printed version of both Form A and Form B of the DOT in a counterbalanced manner. At the top of the page, there is a row of six squares that is numbered from 1 to 6, which serves as a code key for completing this task. There are nine square grids below this, each of which showcases a unique pattern composed of a specific combination of the numbered squares in the code key. Participants complete empty grids below these patterned grids using the corresponding numbered squares from the code key. As it is possible to get a ceiling effect within two minutes, participants were allotted one minute to complete this measure (50). Scores range from 0 to 112. Higher scores indicate better performance.”

**Discussion:**

*1. Line 406f: „it would seem obvious…“ Why would this seem obvious? Please define the discrepancy between the examples further. Is the hypothetical „new environment“ a virtual environment? Are tactile, vestibular, other sensory cues available? Rather than starting with such a vague statement, consider coming back towards the hypotheses from the introduction on what relationships and correlations you assumed based on the literature review. Alternatively, if available, start with studies on real life datasets where people with good performance in one spatial task tend to be good in others, too.*

Apologies for the confusion, and thank you for your suggestions. We have now modified the first paragraph of the discussion section (L476-484) to tie it more closely to our hypotheses from the introduction section based on a review of the literature.

“We tested participants with virtual navigation tasks (wayfinding, PI and RAM) in the gaming app SHQ, visuospatial abilities (mental rotation, visuospatial processing and VSWM), and navigation strategies and preferences (sense of direction, mapping tendency and RAM navigation strategy) to better understand how these cognitive constructs relate to each other. The different constructs showed low levels of association, with negligible correlation among the three spatial navigation tasks on SHQ, and weak correlation between these and the self-ratings and navigation strategies. We observed modest correlations between each of the three visuospatial abilities, and all of them with wayfinding, but not with other navigation tasks. We discuss what these results mean for understanding cognitive profiles of navigation ability.”

*2. Line 425: Please specify that you refer to wayfinding in a mobile game rather than real-world navigation wayfinding , you might reference [15] again here while also please pointing out the methodical differences.*

Thank you for spotting this. We have clarified the use of Sea Hero Quest in L493-496 of the discussion section.

“This is a pattern discussed in past research exploring the relation between small-scale spatial abilities and large-scale wayfinding ability using SHQ and real-world navigation tasks involving various measures such as accuracy, reaction time, distance travelled and number of errors (11).”

*3. Line 453: „In the UK based sample…“ Do you think it’s an UK specific effect? If yes, please add information on why this UK cohort would especially rely on GPS to navigate. Otherwise please rephrase the segment.*

Thank you for pointing this out. We have rephrased this sentence (529-532) in the discussion section

“It is likely that participants use GPS-based systems to find their way, and might rarely find themselves in the situation simulated in the wayfinding task where a map is studied and must be committed to memory before navigation.”

*4. Line 461: The whole analysis of stress level during task performance is not conclusive without information available on baseline stress/anxiety levels. Given the data collection period from 2018-2019, re-evaluation of baseline stress is probably impossible – however, please discuss the absence of baseline stress level testing in its regard to the presented findings.*

Please refer to our second response under the “overall” sub-header for Reviewer 1 Comments.

*5. Line 500: „…at least in a UK university student sample.“ This is the main limitation of the study. This factor should be discussed in regards to all other results: are you observing general effects or is there bias due to the participant cohort?*

Please refer to our first response under the “overall” sub-header for Reviewer 1 Comments.

**REVIEWER 2 COMMENTS AND RESPONSES**

**Overall:**

*I enjoyed reading the manuscript and was curious about the relevant topic of this work. I believe it concerns timely and meaningful data that should be published. The SHQ work is highly impactful and provides a strong and informative foundation for spatial cognition research. However, I also found the manuscript lacking in depth and theoretical detail in several places throughout the text. I would recommend some substantial additions and clarifications throughout the text before recommending publication of this work. Below I have listed my comments in order of appearance in the manuscript:*

Thank you for your detailed feedback. We have substantially revised the manuscript in light of all the recommendations.

**Highlights:**
*The assessment of self-ratings is mentioned without mention of outcome, and the outcomes of strategy are included without mention of its assessment. perhaps pick one of the two for the highlights section*

Thank you for pointing this out. We have edited the first bullet in the highlights section (L37-39).

“Three navigation tests embedded in the game Sea Hero Quest were examined in relation to three object-based visuospatial tasks, and self-ratings of navigation strategy and gameplay stress.”

**Abstract:**

*The main comment that comes to mind in reading the manuscript is that terminology is used quite loosely. I would recommend a more clear and uniform terminology throughout. For instance, self-ratings are labelled as measures of 'confidence' in the abstract, which is a different construct which does not seem to be measured here. The phrase 'those good at wayfinding' is not clear in what group is referred to, those who were specifically good in the subtest of wayfinding of the SHQ? The manuscript would benefit from a thorough check of different terms used throughout. The field already suffers from a large variety of different terms, tasks, questionnaires, etc., it would be very helpful to provide clarity. I will include some examples below.*

Thank you for pointing this out. We have made the terminology uniform throughout the manuscript. We have also edited the abstract to remove the term ‘confidence’, and rephrased L78-82 to specify what we meant by 'those good at wayfinding’ earlier.

“Being good at wayfinding in a virtual navigation test does not mean an individual will also be good at path integration, have a superior memory in a radial arm maze, or rate themself as having a strong sense of direction. However, we observed that participants who were good in the wayfinding task of SHQ tended to perform well on the three visuospatial tasks examined here, and to also use a landmark strategy in the radial maze task.”

**Introduction:**

*The first paragraph could be rewritten. The relevance of specifically including the tasks that the authors selected is not made clear, other than 'variation exists'. Variation exists for all cognitive abilities, so this does not provide any specific explanation. The introduction is very brief and lacks conviction for the task and sample selection in the current study. A related issue is that the tasks and questionnaires are referred to by their names, rather than the cognitive constructs involved. There is ample evidence suggesting that Corsi forward is more related to spatial attention than to spatial working memory for instance. PI is not provided in full the first time. There is no clear research question, the hypothesized correlations are quite elaborate, but not motivated other than in a brief table legend. I was a bit surprised to read that if no existing literature was available, correlations were based on 'general assumptions'. I would really be interested in learning the reasoning behind those assumptions.*

Thank you for your helpful suggestions. We have re-written the first paragraph of the introduction section (please see L89-99). We have also added two detailed paragraphs in the introduction to make it more comprehensive particularly with respect to task selection and cognitive components measured by various tests. These additional paragraphs should also provide some motivation for the hypothesised relationships. Please refer to L122-158 in the introduction. We have also referred to tasks and questionnaires by the cognitive construct they measure, rather than their names, throughout the manuscript. We have provided the full form of PI when we mention it the first time. We have specified our research question and predictions in L171-188. We have also explained our reasoning behind the assumptions we have made in Table 1. Please see L193-200 in Notes under Table 1.

L89-99: “Navigation is a fundamental skill that underlies exploration and survival. The ability to effectively navigate involves a host of capacities such as planning routes, reading maps, recognizing landmarks and keeping track of direction. Deficits in these competencies may constitute an early marker of conditions such as Alzheimer’s disease (AD), multiple sclerosis, vestibular syndrome and autism (1-4). Furthermore, navigation impairments like disorientation can affect one’s quality of life by causing distress and impediments in daily functioning (5, 6). Therefore, understanding individual differences in competencies and strategies underlying navigation can be useful in not only identifying risk factors of cognitive decline, but also in designing tools, interventions and environments to cater to unique navigational needs. More broadly, knowledge of the complex mechanisms and interactions involved in navigation will advance the domain of spatial cognition at large.”

New Para 1 (L122-143): “Navigation performance as assessed by SHQ depends on visuospatial abilities (17-19). These abilities comprise a set of distinct skills involved in perceiving and manipulating objects in space (20-22). One such skill is mental rotation, which involves transforming a mental representation of an object to predict how it would look from different angles (23). Mental rotation facilitates navigation by enabling the formation of precise and flexible mental models of the environment (24), which can be useful to enhance spatial orientation and navigation performance. In addition to the Mental Rotation Test (MRT) (23), we employ the recent Design Organisation Test (DOT) (25) as a measure of visuospatial processing, a cognitive competency involved in navigation (26). A classic test of visuospatial processing is the Block Design sub-test of the Weshler scales, which involves rearranging blocks that have various colour patterns on different sides with the aim of matching a target pattern (27). Previous studies have indicated a strong correlation between this test and neuropsychological measures of visuospatial ability (28) and everyday visuospatial skills (29). Notably, the DOT correlates highly with the Block Design sub-test, but does not involve fine motor skills like in the latter, and is substantially quicker to administer (25). Finally, we also test visuospatial working memory (VSWM) using the forward digital Corsi Block Tapping Test (D-Corsi) as it is another cognitive construct that underlies navigation (30). Corsi performance requires participants to tap spatially separated blocks in a given sequence (31). The two forms of Corsi, forward and backward, have been thought to have different cognitive demands (32). Specifically, it has been suggested that the backward condition assesses executive function and visuospatial short-term memory, whereas the forward condition assesses only the latter (33, 34). Therefore, the forward Corsi test may serve as a more accurate measure of VSWM.”

New Para 2 (L145-158): “Navigational ability depends not only on visuospatial abilities, but also on personal preferences and strategies, which are usually assessed using questionnaires. The type of preferred navigational strategy, as measured by the Navigational Strategies Questionnaire (35), may determine one’s navigational performance. Navigating using a map-based strategy, relative to a non-map-based strategy, for instance, requires a fine-grained and integrated representation of the environment (36). Individuals with a preference for the aforementioned strategy are generally considered to be better at navigation tasks (35), and to show greater flexibility when navigating (37). Additionally, perception of one’s own spatial orientation in the environment may also influence navigation. In fact, an association between performance on tasks requiring participants to update their location in space as a result of self-motion and scores on the SBSOD has been found (38). Overall, the existing literature suggests that visuospatial abilities and navigational preferences and strategies are independent but related factors that contribute to navigation ability (18, 39, 40). Nevertheless, the heterogeneity in testing in existing studies means that the nature of these connections requires further study.”

L171-188: “In this study, we explore how visuospatial abilities, navigation strategy and gameplay stress relate to performance on SHQ and to each other. Based on the literature (e.g., Wolbers & Hegarty (17)), we made 20 predictions. We expected wayfinding to correlate with other measures due to its diverse demands such as perception, memory, decision-making, etc. Specifically, we predicted that longer duration to complete wayfinding levels (i.e., wayfinding inefficiency) would be associated with lower mental rotation, visuospatial processing, VSWM, sense of direction and mapping tendency as measured by the MRT, DOT, D-Corsi, SBSOD and NSQ, respectively. Greater wayfinding inefficiency would also be related to higher total SHQ gameplay duration and SHQ stress. We further predicted that more correct answers on PI levels would be associated with stronger VSWM and sense of direction as measured by D-Corsi and SBSOD, respectively. Moreover, we hypothesized that the number of reference memory errors (i.e., RM errors) and spatial working memory errors (i.e., SWM errors) on RAM levels would positively correlate with each other and negatively with D-Corsi and SBSOD scores. Additionally, we predicted that mental rotation and visuospatial processing, as measured by the MRT and the DOT, respectively, would positively correlate with each other and with VSWM, as measured by the D-Corsi. Our final hypothesis was that sense of direction and mapping tendency, as measured by the SBSOD and the NSQ, respectively, would positively correlate with each other. Table 1 below shows the 20 hypothesised relationships between performance on different tasks.”

L193-200: “*Note.* The predictions shown are based on the existing literature and (in bold) on previous findings from SHQ (Coutrot et al. (8); Hegarty et al. (18); Hegarty et al. (38); Lawton et al. (44); West et al. (45)*.* Where there was no direct research on the relationship(s) between the specific measures used in this study, we relied on research on how various constructs assessed by the measures are associated with each other. For instance, our prediction on the relationship between wayfinding on SHQ and mapping tendency, as measured by the NSQ, is based on the finding that those who use a map-based strategy, relative to a non-map-based strategy, are generally considered to be better at navigation tasks (35). Similarly, we hypothesised that sense of direction, as measured by the SBSOD, will be positively associated with navigation on SHQ given that better sense of direction is usually related to efficient navigation (46, 47). We predicted that gameplay stress will be negatively associated with navigation based on previous research that shows stress impairs wayfinding and path integration performance (48, 49). Lastly, the observation that individuals with high VSWM are faster and make fewer errors during wayfinding influenced our hypothesis about the relationship between SHQ wayfinding and VSWM as indicated by D-Corsi (19).”

**Methods:**

*The sample composition was very skewed in age group and gender, I wondered if this was included in the comparisons that were made with other samples (e.g. for the power analyses) and how the authors think this might affect their outcomes*. *The level selections were not motivated, there is mention of difficulty etc., but not why those particular levels of difficulty were worth selecting. The stress measurement seems rather limited, as this was a lab study, some individuals could have just been stressed about the lab setting, rather than the navigation task at hand, or some prior event right before the assessment, is there any measure of pre-test stress level available? If not, this seems important to include in the discussion. Both DOT and Corsi are not described based on the cognitive constructs measured. This adds to the confusion about the different terms used throughout the manuscript. Why was the Corsi backward not included?*

Thank you for your input. We have written about the implications of having a skewed sample in the limitations paragraph of the discussion section (please see L567-573); this will be an interesting avenue to explore in future work. We have also provided motivation for the selection of the chosen levels in L233-236. Unfortunately, due to a technical error at the time of data collection, the baseline stress and mood ratings of participants could not be recorded. We have now clarified as such in the discussion section, and have also written the implications of the absence of baseline stress levels. Please refer to L542-546 in the discussion section. In line with your suggestions, we have reworded the entire discussion section to mention all the questionnaires and tasks in terms of the construct they measure instead of their names; please see pages 26-30 (which are also mentioned below in our response to comments under the “discussion” sub-header of Reviewer 2 Comments). We also have clarified why Corsi forward was included in this study; please see L136-143 in the introduction section.

L567-573: “Relatedly, the sample was overwhelmingly composed of young and female participants, which could impact the external validity of the findings. Since previous research has shown that differences based on sex, age and culture exist in navigation performance and related behaviours, it would be helpful to use large samples to explore a broader range of ages, and compare gender and performance across nations in the future (7). Nonetheless, our results elucidate the relationship among object-based spatial ability, navigation strategy and virtual navigation performance specifically for young women.”

L233-236: “Based on the aforementioned criteria, we chose levels 16, 37, 32 and 43 for wayfinding to keep a broader selection of levels in terms of spatial characteristics of the environment. For PI, we selected levels 44, 49, 54, 59, 64, 69 and 74 that were later in the game and more challenging.”

L542-546: “It is worth mentioning that the baseline stress and mood ratings of participants could not be recorded due to a technical error. Hence, it is possible that SHQ stress ratings were not an accurate measure of the stress caused by the navigation tasks; the stress may have simply been a result of being in a laboratory setting, or a prior event before the assessment.”

L136-143: “Finally, we also test visuospatial working memory (VSWM) using the forward digital Corsi Block Tapping Test (D-Corsi) as it is another cognitive construct that underlies navigation (30). Corsi performance requires participants to tap spatially separated blocks in a given sequence (31). The two forms of Corsi, forward and backward, have been thought to have different cognitive demands (32). Specifically, it has been suggested that the backward condition assesses executive function and visuospatial short-term memory, whereas the forward condition assesses only the latter (33, 34). Therefore, the forward Corsi test may serve as a more accurate measure of VSWM.”

**Discussion**

*I would highly recommend rewording the discussion based on the constructs measured rather than the names of all the different tasks, questionnaires, etc involved, at this point those names only have limited importance and a conceptual discussion would really help understanding the text.*

We agree with your suggestions, and have reworded the discussion section accordingly. Please see pages 26-30.

“We tested participants with virtual navigation tasks (wayfinding, PI and RAM) in the gaming app SHQ, visuospatial abilities (mental rotation, visuospatial processing and VSWM), and navigation strategies and preferences (sense of direction, mapping tendency and RAM navigation strategy) to better understand how these cognitive constructs relate to each other. The different constructs showed low levels of association, with negligible correlation among the three spatial navigation tasks on SHQ, and weak correlation between these and the self-ratings and navigation strategies. We observed modest correlations between each of the three visuospatial abilities, and all of them with wayfinding, but not with other navigation tasks. We discuss what these results mean for understanding cognitive profiles of navigation ability.

Consistent with our predictions, we found a significant correlation between wayfinding performance and performance on visuospatial tasks of mental rotation, visuospatial processing and VSWM. To our knowledge, this is the first study to explore the relationship between visuospatial processing as measured by the DOT and navigation tasks. In all three cases, a low-to-moderate correlation is in line with previous studies examining the relationship between large-scale and small-scale spatial abilities (18, 21). The absence of strong correlations between these abilities indicates that while wayfinding and these constructs may have some overlap in the cognition required, they also have different demands. This is a pattern discussed in past research exploring the relation between small-scale spatial abilities and large-scale wayfinding ability using SHQ and real-world navigation tasks involving various measures such as accuracy, reaction time, distance travelled and number of errors (11). These findings are useful in highlighting the potential utility of VR-based navigation tasks in capturing something that is distinct from object-based visuospatial tasks. One factor that may differentiate wayfinding from the other visuospatial tasks is the reliance on broader executive functions demands. Unlike object-based visuospatial tasks, wayfinding places specific demands on planning and inhibition (54, 55). In SHQ, this involves avoiding re-approaching visited checkpoints and planning optimal paths given the order of checkpoints indicated on the initially shown map.

Interestingly, the three tests of visuospatial ability have a moderate correlation with each other, indicating that they measure different but related cognitive aspects. Mental rotation may require flexible switching between motor simulation and analytic thinking, depending on the difficulty level (56). Block tapping may involve control processes, visual working memory and visuospatial attention (57, 58). Visuospatial processing on the DOT may rely on both visuospatial abilities and problem-solving skills (59). This may explain why visuospatial processing on the DOT has the strongest correlation with wayfinding performance, considering the link between wayfinding performance and executive functions.

We had predicted that performance on RAM levels and VSWM as measured by D-Corsi performance would be correlated because both require holding a set of locations in mind over many seconds i.e., they test spatial working memory. We found no evidence for this. This result is consistent with the view that ‘spatial working memory’ is not a unitary cognitive function, but depends on the context (e.g., 2D screen space versus VR-rendered 3D environment). Theoretically, it seems plausible that the D-Corsi is supported mainly via frontoparietal circuits, and that the RAM additionally draws on hippocampal circuits to support the representation of the large-scale environment (45, 60). The absence of a correlation between the RAM and other navigation tasks further indicates that different cognitive demands between the tasks are involved.

A surprising result was that neither sense of direction nor mapping tendency was correlated with navigation performance on any SHQ task. This stands in contrast with previous evidence of correlations between navigation behaviour and these constructs (35, 38, 61), but is similar to some other evidence (62). It may be that participants need to physically move or use body-based cues to navigate in a space for a stronger association of navigation behaviours with standardised measures such as the SBSOD (e.g., Hegarty et al. (18)). Another possibility is that people tend to rate their navigation ability in relation to how often they might get lost. It is likely that participants use GPS-based systems to find their way, and might rarely find themselves in the situation simulated in the wayfinding task where a map is studied and must be committed to memory before navigation. When we recently sampled a large population, drawn from many nations and a range of ages, we found a consistent relationship between wayfinding performance in SHQ and self-rated navigation ability (9). Thus, the relationship between self-ratings and wayfinding performance is likely moderated by a wide range of factors (e.g., He & Hegarty (63), Hegarty et al. (18), van der Ham & Koutzmpi (64), van der Ham et al. (65)). Notably, while actual performance in the wayfinding task in SHQ was not correlated with sense of direction or mapping tendency, the stress ratings of playing SHQ were, even though they did not reach the stringent Bonferroni-corrected significance level. Thus, people who think they are good navigators, or those who tend to think using maps, might be less likely to find SHQ stressful. In future, it will be useful to explore more about spatial anxiety in daily life and SHQ performance to understand if they are linked more than the stress reported (44, 66). It is worth mentioning that the baseline stress and mood ratings of participants could not be recorded due to a technical error. Hence, it is possible that SHQ stress ratings were not an accurate measure of the stress caused by the navigation tasks; the stress may have simply been a result of being in a laboratory setting, or a prior event before the assessment.

We found that the SBSOD and the NSQ are moderately correlated with each other, which indicates that having a strong sense of direction is associated with using map-based strategy to navigate. To the authors’ knowledge, the association between these two constructs has been explored for the first time. This finding indicates that the two tests capture related dimensions of navigational profiles and attitudes, but not so overlapping as to be equivalent.

We found that those who used the landmark strategy on RAM levels performed significantly better on both RAM and wayfinding levels than those who used the counting strategy. This mirrors recent evidence from the large participant group of over 37,000 participants (45). By combining the NSQ with the RAM tests for this study we reveal, for this population, that landmark-counting strategies appear to be orthogonal to the survey-route dimension captured by the NSQ. Notably, the lack of a correlation between wayfinding inefficiency and the RAM errors in this small lab sample matches our recent report of an absence of correlation between these in an online sample of over 37,000 participants (45). The absence of a correlation of the PI measure and the other navigation tasks is notable in light of recent evidence that PI, rather than other spatial tests, might be particularly important for early detection of AD (67-69).

There are a number of limitations to our study that should be considered. First, our sample size was limited by the challenge of conducting in lab testing, which was central to our planned design. Relatedly, the sample was overwhelmingly composed of young and female participants, which could impact the external validity of the findings. Since previous research has shown that differences based on sex, age and culture exist in navigation performance and related behaviours, it would be helpful to use large samples to explore a broader range of ages, and compare gender and performance across nations in the future (7). Nonetheless, our results elucidate the relationship among object-based spatial ability, navigation strategy and virtual navigation performance specifically for young women. Second, the visuospatial tasks we used here were adapted from clinically used tests aimed at detecting differences between groups, whereas our aim was to explore variation in the population. Thus, it may be useful to further develop such tests to help optimally explore individual differences (70). Finally, as with all correlative approaches to assessing individual differences, the capacity of the tests used will depend partly on the variance with the data generated by the test in the group tested. In our current study, the PI task had less variability than others, and thus, this may have impacted our capacity to detect some relationship between PI and other measures. Nonetheless, we were able to observe predicted correlation between this test and the D-Corsi, indicating the variation present was still sufficient to detect some effects.

In conclusion, our study highlights that many tests and self-rating scales for navigation and visuospatial abilities can be highly non-overlapping, at least in a UK university student sample. Our results help further characterise what the different tests used in SHQ are related to. For example, we show that wayfinding has some overlap with object-based visuospatial tasks, whereas PI and RAM tasks have much less. In future research it would be useful to probe a broader range of environments to understand how the complexity of the layouts (12) and landmark density (71, 72) lead to higher or lower correlations with object-based visuospatial ability.”

**Conclusion:**
*A main source of the limited overlap between measures would most likely be the highly specific task selection, in my opinion, which has not been motivated in this text. If the authors aimed to provide a comprehensive set of measures, this could be problematic, but from the introduction right now, this does not seem to be the case. It would be really helpful to flesh out the exact constructs across the measures, to understand the limited correlations. The future recommendations could be highly interesting but are not motivated here, it would be really helpful to provide a clear motivation for them.*

We have now edited the manuscript to mention all the questionnaires and tasks in terms of the construct they measure instead of their names, and have provided reasoning in the introduction section as to their selection. Please refer to the two newly added paragraphs in the introduction section (L122-158), which are also mentioned in our response to comments under the “introduction” sub-header for Reviewer 2 Comments. We have stuck to the recommendations that are motivated by the study in a clearer fashion, and deleted the rest.

L584-591: “In conclusion, our study highlights that many tests and self-rating scales for navigation and visuospatial abilities can be highly non-overlapping, at least in a UK university student sample. Our results help further characterise what the different tests used in SHQ are related to. For example, we show that wayfinding has some overlap with object-based visuospatial tasks, whereas PI and RAM tasks have much less. In future research it would be useful to probe a broader range of environments to understand how the complexity of the layouts (12) and landmark density (71, 72) lead to higher or lower correlations with object-based visuospatial ability.”
