# Supplementary material for: The relationship between object-based spatial ability and virtual navigation performance
Source: PLoS One. 2024 May 9;19(5):e0298116. doi: 10.1371/journal.pone.0298116 (PMC11081363; doi:10.1371/journal.pone.0298116)
Supplement: Attachment — Submitted filename: ResponseToReviewers.docx [file pone.0298116.s005.docx]

**REPLY TO REVIEWS:**

We are grateful to have the opportunity to reply to the reviews and re-submit.

Below we quote comments from the Editor and Reviewers in ***italics***, our response in **blue** font and quotes from our updated manuscript in **red** font (as in the red font in the re-submitted manuscript).

**EDITOR COMMENTS:**

*There are only a few very minor comments by the reviewers. I thought it would be best to amend the document accordingly before sending it to publication. I will not send the document to be reviewed again.*

Thank you for giving us the opportunity to revise the manuscript. We have addressed the minor comments made by the reviewers.

**REVIEWER 1 COMMENTS:**

**Overall:**

*The revision addressed most of the concerns by both reviewers. Some aspects that would have improved the manuscript have now been mentioned as relevant limitations in the discussion section; sadly, no corrections for baseline stress levels were performed due to data unavailability. The method section is now improved and better readable, some relevant information on testing procedures has been added. Overall, the readability and scope of the manuscript has been improved while the basic limitation of a small, predominantly young and female sample size remains, but is discussed now.

All in all, I still have some minor comments on the text which should be corrected before publication, but they don't require another round of peer review.*

Thank you for your comments!

**Introduction:** *Line 91-93: spatial competency is NOT an early marker of autism or vestibular syndromes, but a possible symptom/another affected domain. Instead, the lines could read "Deficits in these competencies may constitute an early marker of degenerative conditions such as Alzheimer’s disease (AD); furthermore, spatial abilities are impaired in various neurological disorders or conditions, such as multiple sclerosis, vestibular syndromes and autism (1-4)."*

Thank you for pointing out this nuance. We have included the suggested phrasing in the manuscript (pg. 4, L91-94).

“Deficits in these competencies may constitute an early marker of degenerative conditions such as Alzheimer’s disease (AD); furthermore, spatial abilities are impaired in various neurological disorders or conditions, such as multiple sclerosis, vestibular syndromes and autism (1-4).”

*116 ...sleeping 7 hrs per night" compared to what other group?*

We have now included information about the comparison groups (pg. 6, L116-117).

“Such studies have also shown that performance is best for older participants who report sleeping 7 hours per night compared to those reporting more or less sleep (13).”

*Thanks to the authors for addressing the reviewers comments.*

We thank you for your thoughtful comments!

**REVIEWER 2 COMMENTS:**

**Overall:**

*The revision has been thorough, I appreciate the authors' efforts to incorporate all issues raised. I have two minor points that I would recommend to address and one comment I would like to share:*

**Abstract:**

*In the final sentence, the authors indicate 'support', which would imply a causal relationship between the variables mentioned, I would suggest to avoid such implication as this was not tested.*

Thank you for raising this point. We have now rephrased this sentence (pg. 4, L80-81).

“These findings help clarify the associations between different abilities involved in spatial navigation.”

**Conclusion:**

*This is much clearer now. I think the neuropsychological literature concerning navigation ability could be used here to further support this conclusion. It is notably difficult to try to capture navigation performance with standardized testing materials concerning spatial cognition, your results highlight the mostly likely reasons for this. Most likely, the distinction between small and large scale spatial functioning (only partially overlapping), is the central cue here. Perhaps the authors could incorporate this connection (to limitations of standardized testing materials, and small vs large scale functioning) to further strengthen their position.*

Thank you for this suggestion. We have added a sentence about this in the conclusion (pg. 30, L590-592).

“The findings also point to the limitations of standardised tests of spatial cognition in capturing the nuances of navigation performance, particularly the distinction between small- and large-scale functioning.”

**Final Thoughts:**

*The digital version of the Corsi is conceptually different from the regular manual Corsi task. Here, the predictive finger movements are absent, which result in slightly different processing and performance.*

Thank you for sharing your thoughts with us. We have added the following clarification under the “Digital Corsi” sub-header in the methodology section (pg. 15, L303-305).

“It is important to note the digital version of the Corsi task is conceptually different from the regular manual version. Specifically, in the digital version, the absence of predictive finger movements results in slightly different processing and performance.”
